# Sphingosine kinase 1 contributes to the metastatic potential of epithelial ovarian cancer to the adipocyte-rich niche

**DOI:** 10.1186/s40164-022-00358-y

**Published:** 2022-11-16

**Authors:** Chen Wang, Taiyang Ye, Wenjing Wang, Keqi Song, Jie Zhu, Lan Dai, Wen Di

**Affiliations:** 1 Department of Obstetrics and Gynecology, Ren Ji Hospital, School of Medicine, Shanghai Jiao Tong University, Shanghai, 200127 China; 2 Shanghai Key Laboratory of Gynecologic Oncology, Ren Ji Hospital, School of Medicine, Shanghai Jiao Tong University, Shanghai, 200127 China; 3 State Key Laboratory of Oncogenes and Related Genes, Shanghai Cancer Institute, Ren Ji Hospital, School of Medicine, Shanghai Jiao Tong University, Shanghai, 200127 China

**Keywords:** Sphingosine kinase 1 (SphK1), Adipocyte, Ovarian cancer, Metastasis

## Abstract

Unlike many solid tumors, epithelial ovarian cancer (EOC) has a clear metastatic predilection to the adipocyte-rich niche, especially the omentum. However, the underlying mechanism driving this process remains incomplete. Here we show that SphK1 is over-expressed in omental metastases compared with ovarian primary tumors in EOC patients. In vitro, inhibition of SphK1 suppressed the metastatic ability of EOC induced by adipocytes. In vivo, blockage of SphK1 could attenuate the omental metastasis of EOC. Importantly, SphK1 modulates adipocyte-induced E/N-cadherin switch through Twist1, a key process in EOC metastasis. Our study reveals a previously unrecognized role of SphK1 in modulating the metastatic tropism of EOC to the adipocyte-rich niche, suggesting a new target for EOC therapy.

**Supplementary Information:**

The online version contains supplementary material available at 10.1186/s40164-022-00358-y.

## To the editor,

Epithelial ovarian cancer (EOC) is the deadliest gynecological malignancy [1]. Unlike many solid tumors, EOC tends to metastasize to the adipocyte-rich microenvironment, especially the omentum [2, 3]. This metastatic tropism of EOC can lead to extensive pelvic and abdominal metastasis, which is the major cause of death in EOC patients. Therefore, identifying the mechanisms driving this process is crucial for disease-specific therapeutic intervention. Sphingosine kinase 1 (SphK1) is a key enzyme with a well-established role in the regulation of sphingolipid metabolism [4]. SphK1 catalyzes the phosphorylation of sphingosine to produce sphingosine-1-phosphate (S1P), which can act through S1P receptor (S1PR) pathway and intracellular pathway [4]. We previously showed that SphK1 was over-expressed in EOC tissue and was involved in EOC growth and angiogenesis [5–7]. We recently found adipocytes could activate SphK1 in EOC cells [6]. In this work, we tested the role of SphK1 in EOC metastasis induced by adipocyte-rich niche.

**Fig. 1:**
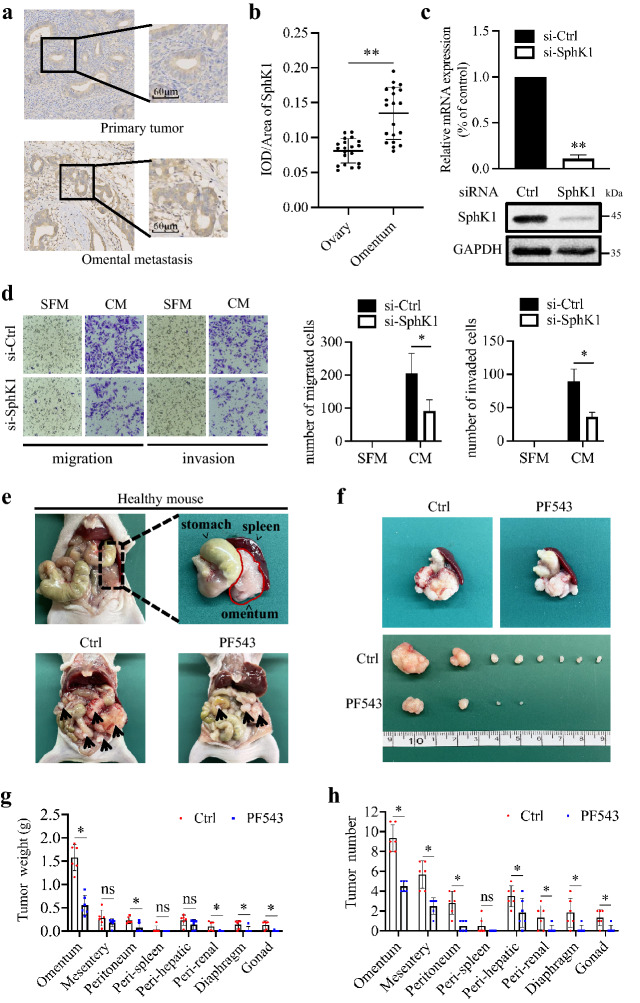
Inhibition of SphK1 suppressed EOC metastasis to the adipocyte-rich niche. **a** SphK1 IHC of paired samples from ovarian primary tumors and their corresponding metastatic omental deposits from 20 patients. Representative images are shown. The scale bar represents 60 μm. **b** Quantitative analysis of IHC staining for SphK1 of ovarian primary tumors and their corresponding metastatic omental deposits from 20 patients. **P < 0.01; two-tailed Student’s *t* test. **C** SKOV3 cells were transfected with negative control siRNA (si-Ctrl) or SphK1 siRNA (si-SphK1). 24 h after transfection, mRNA level of SphK1 was determined by quantitative reverse transcription polymerase chain reaction (qRT-PCR) and normalized to GAPDH. 48 h after transfection, protein level of SphK1 was determined by Western blot and normalized to GAPDH. Data are mean ± SD (*n* = 3). **P < 0.01; two-tailed Student’s *t* test. **d** Representative images of migration and invasion assays (200×). Transfected SKOV3 cells were serum starved overnight and metastasized towards serum free medium (SFM) or adipocyte CM. Migrated cells and invaded cells were photographed and calculated. Experiments have been carried out with adipocytes from 2 to 3 independent donors. Data are mean ± SD (*n* = 3). *P < 0.05; two-tailed Student’s *t* test. **e** Representative images of omentum in healthy mouse and representative images of EOC xenograft mouse model in the Ctrl and PF543 group. Black arrows indicated the location of the metastatic nodules. **f** Representative images of omental metastatic tumors in EOC mouse models of the Ctrl and PF543 group. **g** Quantification of tumor weight in different metastatic sites. Data are mean ± SD (*n* = 6). *P < 0.05 versus Ctrl group; ns, no significance; two-tailed Student’s *t* test. **h** Quantification of tumor number in different metastatic sites. Data are mean ± SD (*n* = 6). *P < 0.05 versus Ctrl group; ns, no significance; two-tailed Student’s t-test

We first performed SphK1 immunohistochemistry (IHC) on paired samples from ovarian primary tumor and omental metastatic tissue of 20 high-grade serous ovarian cancer patients (Additional file 1: Table S1). Quantification confirmed that SphK1 expression was significantly increased in omental metastatic deposits compared with the primary tumors (Fig. 1a, b). Because adipocytes, major components of the omentum, promotes the metastasis of EOC [8], we next tested whether SphK1 was involved in EOC metastasis induced by adipocytes from human omentum (Additional file 2: Fig. S1A). Markedly, blockage of SphK1 by either siRNA or selective inhibitor PF543 significantly inhibited SKOV3 cell migration and invasion induced by adipocyte culture medium (CM) (Fig. 1c, d; Additional file 3: Fig. S2A–D). We used Hey cells, another EOC cell line, to confirm our results (Additional file 2: Fig. S1B–E, Additional file 3: Fig. S2A–D). To test whether SphK1 is required for the omental metastasis, we established xenograft mouse models of EOC by intraperitoneally injecting human EOC cell line SKOV3. Then the mice were injected with PF543 intraperitoneally twice a week. We found that the largest tumor is often formed in the omentum. PF543 treatment resulted in less tumor burden in the omentum and other metastatic sites compared with the control group (Fig. 1e, f). We confirmed this observation by measuring and calculating the tumor weight and the tumor number of metastatic nodules (Fig. 1g, h). We also used SphK1 knockdown EOC cells to confirm our results (Additional file 6: Fig. S5A–E). Together, these results suggested that SphK1 contributed to EOC metastasis to the adipocyte-rich niche.

**Fig. 2:**
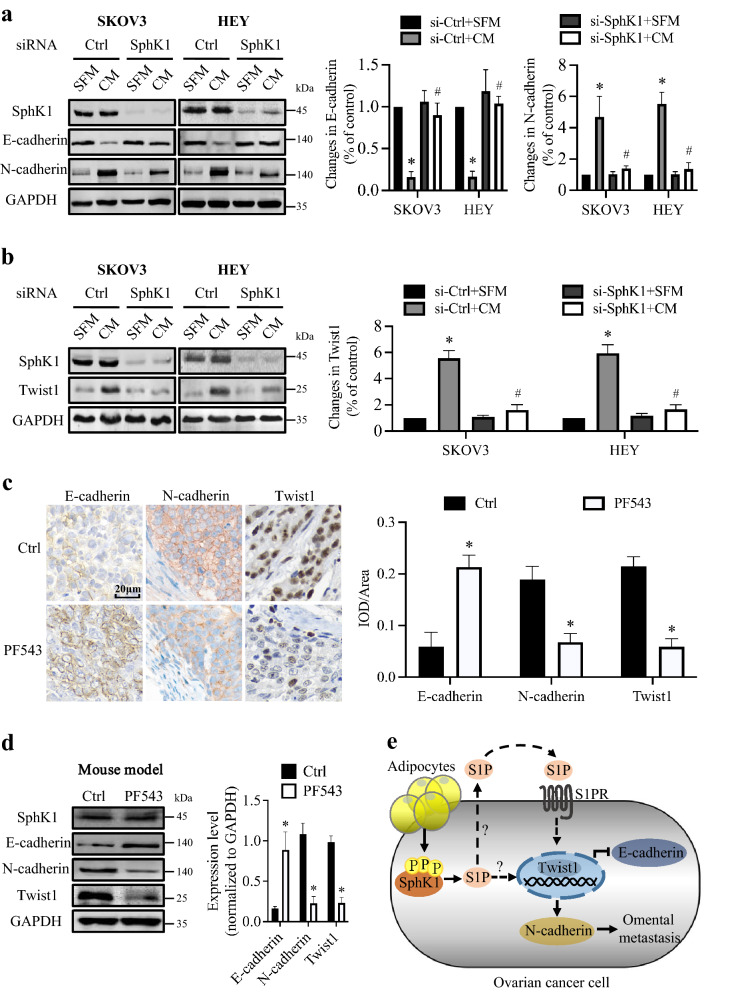
SphK1 modulates adipocyte-induced E/N-cadherin switch through Twist1. **a** SKOV3 and HEY cells were transfected with negative control siRNA or SphK1 siRNA. Cells were serum starved overnight and cultured in SFM or adipocyte CM for 48 h. Expression levels of SphK1, E-cadherin and N-cadherin were determined by Western blot and normalized to GAPDH. Densitometric analyses of E-cadherin and N-cadherin were shown on the right. Experiments have been carried out with adipocytes from 2 to 3 independent donors. Data are mean ± SD (*n* = 3). *P < 0.05 versus control; ^#^*P* < 0.05 versus adipocyte CM alone; two-tailed Student’s *t* test. **b** SKOV3 and HEY cells were transfected with negative control siRNA or SphK1 siRNA. Cells were serum starved overnight and cultured in SFM or adipocyte CM for 48 h. Expression level of SphK1 and Twist1 was determined by Western blot and normalized to GAPDH. Densitometric analysis of Twist1 was shown on the right. Experiments have been carried out with adipocytes from 2 to 3 independent donors. Data are mean ± SD (*n* = 3). *P < 0.05 versus control; ^#^*P* < 0.05 versus adipocyte CM alone; two-tailed Student’s *t* test. **C** IHC staining of E-cadherin, N-cadherin and Twist1 in the omental metastatic tumor tissue of mouse models. The scale bar represents 20 μm. Statistical analysis of integrated optical density (IOD)/area was shown on the right. Data are mean ± SD (*n* = 6). *P < 0.05 versus Ctrl group; two-tailed Student’s *t*-test. **d** Expression levels of SphK1, E-cadherin, N-cadherin and Twist1 in omental metastatic tumor tissue of mouse models were determined by Western blot and normalized to GAPDH. Densitometric analyses of E-cadherin, N-cadherin and Twist1 were shown on the right. Data are mean ± SD (*n* = 6). *P < 0.05 versus Ctrl group; two-tailed Student’s *t* test. **e** Model of adipocyte-induced omental metastasis of EOC cells. Adipocytes are capable of activating SphK1, leading to the release of S1P. SphK1/S1P signaling may subsequently activate Twist1 through S1P receptor (S1PR) pathway and/or intracellular pathway, which drives E/N-cadherin switch

Epithelial-mesenchymal transition (EMT) was recognized as a key process in tumor metastasis [9]. EMT was characterized by decreased expression of epithelial cadherin (E-cadherin) and increased expression of neural cadherin (N-cadherin), referred to as ‘E/N-cadherin switch’ [10]. In EOC, E/N-cadherin switch is associated with EMT and invasive phenotype acquisition [11]. Because adipocytes promoted EOC metastasis, we investigated whether adipocytes regulated E/N-cadherin switch. As expected, we found that adipocyte CM induced the gain of N-cadherin accompanied by the loss of E-cadherin in EOC (Fig. 2a). Having shown the function of SphK1 in adipocyte-induced metastasis of EOC, potential roles of SphK1 in adipocyte-induced E/N-cadherin switch was suggested. Indeed, SphK1 inhibition significantly attenuated the level change of E-cadherin and N-cadherin induced by adipocytes (Fig. 2a). These results indicated an important role of SphK1 in regulating adipocyte-induced E/N-cadherin switch. E-cadherin and N-cadherin expression is notably controlled by zinc-finger transcriptional factors, including Snail, Slug, Twist1 and ZEB [12]. Therefore, we further invested the role of adipocytes in these factors. Expression level of Twist1 (Fig. 2b), but not Snail, Slug, ZEB1 or ZEB2 (Additional file 4: Fig. S3A, B), was enhanced in EOC by adipocyte CM co-culture, which indicated that adipocyte may affect E/N-cadherin switch in EOC through Twist1 activation. Moreover, SphK1 blockage significantly attenuated the adipocyte-induced Twist1 expression in EOC (Fig. 2b). Furthermore, suppression of Twist1 by siRNA could significantly inhibit EOC cell migration and invasion induced by adipocyte CM (Additional file 5: Fig. S4A–D). In addition, SphK1 blockage enhanced E/N-cadherin switch and inhibited Twist1 expression in mouse omental metastases models (Fig. 2c, d; Additional file 6: Fig. S5F, G). Together, these results suggest that SphK1 is involved in adipocyte-induced Twist1 activation, which subsequently drives the E/N-cadherin switch.

In conclusion, our study provided mechanistic data delineating a new mechanism that mediated the metastatic potential of EOC to the adipocyte-rich niche via SphK1 signaling (Fig. 2e), suggesting a new target for EOC therapy.

## Supplementary Information

**Additional file 1: Table S1.**

**Additional file 2: Fig. S1** Inhibition of SphK1 by siRNA suppressed adipocyte-induced metastasis of HEY cells. **A** Adipocyte isolation. Primary human omentum tissues were collected and mature adipocytes were extracted. Adipocytes were visualized by (i) microscopy, (ii) stained with Oil red O to confirm the extraction of mature adipocytes, and (iii) by fluorescence microscopy with calcein-AM to confirm viability. The scale bar represents 100 μm. **B** HEY cells were transfected with negative control siRNA or SphK1 siRNA. 24 h after transfection, mRNA level of SphK1 was determined by qRT-PCR and normalized to GAPDH. Data are mean ± SD (*n* = 3). **P < 0.01; two-tailed Student’s *t* test. **C** 48 h after transfection, protein level of SphK1 was determined by Western blot and normalized to GAPDH. Densitometric analysis of SphK1 was shown on the right. Data are mean ± SD (*n* = 3). **P < 0.01; two-tailed Student’s *t* test. **D** Representative images of migration assay (200×). Transfected HEY cells were serum starved overnight and migrated towards SFM or adipocyte CM. Migrated cells were photographed and calculated. Experiments have been carried out with adipocytes from 2 to 3 independent donors. Data are mean ± SD (*n* = 3). *P < 0.05; two-tailed Student’s t-test. **E** Representative images of invasion assay (200×). Transfected HEY cells were serum starved overnight and invaded towards SFM or adipocyte CM. Invaded cells were photographed and calculated. Experiments have been carried out with adipocytes from 2 to 3 independent donors. Data are mean ± SD (*n* = 3). *P < 0.05; two-tailed Student’s *t* test

**Additional file 3:** **Fig. S2** SphK1 inhibitor PF543 suppressed adipocyte-induced metastasis of EOC cells. **A** Serum-starved SKOV3 and HEY cells were treated with increasing doses of PF543 as indicated for 2 h. Expression level of SphK1 was determined by Western blot and normalized to GAPDH. Densitometric analysis of SphK1 was shown on the right. Data are mean ± SD (*n* = 3). ns, no significance versus control; two-tailed Student’s *t* test.** B** Serum-starved SKOV3 and HEY cells were treated with 10 μM PF543 for the indicated time. Expression level of SphK1 was determined by Western blot and normalized to GAPDH. Densitometric analysis of SphK1 was shown on the right. Data are mean ± SD (*n* = 3). ns, no significance versus control; two-tailed Student’s *t* test. **C** Representative images of migration assay (200×). SKOV3 and HEY cells were serum starved overnight, pretreated with PF543 (10 μM) for 2 h, and then migrated towards SFM or adipocyte CM. Migrated cells were photographed and calculated. Experiments have been carried out with adipocytes from 2 to 3 independent donors. Data are mean ± SD (*n* = 3). *P < 0.05; two-tailed Student’s *t* test. **D** Representative images of invasion assay (200×). SKOV3 and HEY cells were serum starved overnight, pretreated with PF543 (10 μM) for 2 h, and then invaded towards SFM or adipocyte CM. Invaded cells were photographed and calculated. Experiments have been carried out with adipocytes from 2 to 3 independent donors. Data are mean ± SD (*n* = 3). *P < 0.05; two-tailed Student’s *t* test

**Additional file 4: Fig. S3** Effect of adipocyte CM on the expression level of Snail, Slug, ZEB1 and ZEB2 in EOC cells. **A** SKOV3 cells were serum starved overnight and cultured in SFM or adipocyte CM for 48 h. Expression levels of Snail, Slug, ZEB1 and ZEB2 were determined by Western blot and normalized to GAPDH. Densitometric analyses were shown on the right. Experiments have been carried out with adipocytes from 2 to 3 independent donors. Data are mean ± SD (*n* = 3). ns, no significance; two-tailed Student’s *t* test. **B** HEY cells serum starved overnight and cultured in SFM or adipocyte CM for 48 h. Expression levels of Snail, Slug, ZEB1 and ZEB2 were determined by Western blot and normalized to GAPDH. Densitometric analyses were shown on the right. Experiments have been carried out with adipocytes from 2 to 3 independent donors. Data are mean ± SD (*n* = 3). ns, no significance; two-tailed Student’s *t* test

**Additional file 5: Fig. S4** Inhibition of Twist1 by siRNA suppressed adipocyte-induced metastasis of EOC cells. **A** SKOV3 and HEY cells were transfected with negative control siRNA (si-Ctrl) or Twist1 siRNA (si-Twist1). 24 h after transfection, mRNA level of Twist1 was determined by qRT-PCR and normalized to GAPDH. Data are mean ± SD (*n* = 3). **P < 0.01; two-tailed Student’s *t*-test. **B** 48 h after transfection, protein level of Twist1 was determined by Western blot and normalized to GAPDH. **C** Representative images of migration assay (200×). Transfected cells were serum starved overnight and migrated towards SFM or adipocyte CM. Migrated cells were photographed and calculated. Experiments have been carried out with adipocytes from 2 to 3 independent donors. Data are mean ± SD (*n* = 3). *P < 0.05; two-tailed Student’s *t*-test. **D** Representative images of invasion assay (200×). Transfected cells were serum starved overnight and invaded towards SFM or adipocyte CM. Invaded cells were photographed and calculated. Experiments have been carried out with adipocytes from 2 to 3 independent donors. Data are mean ± SD (*n* = 3). *P < 0.05; two-tailed Student’s *t*-test

**Additional file 6: Fig. S5** Inhibition of SphK1 suppressed omental metastasis of EOC in vivo. **A** Expression of SphK1 in stable transfected SKOV3 cells tested by qRT-PCR and Western blot. GAPDH was used as a loading control. Data are mean ± SD (*n* = 3). *P < 0.05 versus Blank; ns, no significance versus Blank; two-tailed Student’s *t*-test. **B** Representative images of disseminated tumors in intraperitoneal EOC xenograft mouse models injected with Ctrl shRNA (sh-Ctrl) transfected or SphK1 shRNA (sh-SphK1) transfected SKOV3 cells. Black arrows indicated the location of the metastatic nodules.** C** Representative images of omental metastatic tumors in mouse models. **D** Quantification of tumor weight in different metastatic sites. Data are mean ± SD (*n* = 6). *P < 0.05 versus sh-Ctrl group; ns, no significance; two-tailed Student’s *t* test. **E** Quantification of tumor number in different metastatic sites. Data are mean ± SD (*n* = 6). *P < 0.05; ns, no significance; two-tailed Student’s *t* test. **F** IHC staining of E-cadherin, N-cadherin and Twist1 in omental metastatic tumor tissue of mouse models. The scale bar represents 20 μm. Statistical analysis of IOD/area was shown on the right. Data are mean ± SD (*n* = 6). *P < 0.05 versus sh-Ctrl group; two-tailed Student’s *t*-test. **G** Expression levels of E-cadherin, N-cadherin and Twist1 in omental metastatic tumor tissue were determined by Western blot and normalized to GAPDH. Densitometric analyses of E-cadherin, N-cadherin and Twist1 were shown on the right. Data are mean ± SD (*n* = 6). *P < 0.05 versus sh-Ctrl group; two-tailed Student’s t-test

**Additional file 7:** Supplementary Methods.

## Data Availability

Supplementary information including Additional files 1, 2, 3, 4. 5, 6, 7 are provided with the online version of this paper. The datasets analyzed during the current study are available from the corresponding author on reasonable request.

## Abbreviations

EOC: Epithelial ovarian cancer
SphK1: Sphingosine kinase 1
S1P: Sphingosine-1-phosphate
S1PR: Sphingosine-1-phosphate receptor
IHC: Immunohistochemistry
siRNA: Small interfering RNA
CM: Culture medium
PBS: Phosphate buffered saline
EMT: Epithelial-mesenchymal transition
E-cadherin: Epithelial cadherin
N-cadherin: Neural cadherin
qRT-PCR: Quantitative reverse transcription polymerase chain reaction
SFM: Serum free medium
IOD: Integrated optical density
