# Supplementary material for: Sphingosine kinase 1 contributes to the metastatic potential of epithelial ovarian cancer to the adipocyte-rich niche
Source: Exp Hematol Oncol. 2022 Nov 16;11:102. doi: 10.1186/s40164-022-00358-y (PMC9667684; doi:10.1186/s40164-022-00358-y)
Supplement: Supplementary file 1 — Additional file 1: Table S1. [file 40164_2022_358_MOESM1_ESM.docx]

Table S1: Clinical data on the Serous ovarian cancer, Related to Fig.1

Histopathological and clinical data on the 20 serous ovarian cancer patients. The following information is listed in order across the columns: (1) patient number; (2) patient age at inclusion (years); (3) FIGO stage; (4) tumor grade; (5) CA125 at diagnosis (kU/L); (6) HE4 at diagnosis (pmol/L); (7) p53 staining by immunohistochemistry; (8) BRCA status by genetic testing and (9) tumor metastatic site.

| **Patient** | **Age at inclusion (y)** | **FIGO Stage** | **Tumor Grade** | **CA125 at diagnosis (kU/L)** | **HE4 at diagnosis (pmol/L)** | **p53 (IHC)** | **BRCA status** | **Metastatic site** |
| --- | --- | --- | --- | --- | --- | --- | --- | --- |
| P1 | 45 | III | High-grade | 880 | 401 | mutant IHC pattern | *BRCA1* deleterous mutation | uterine adnexa, rectum, omentum, colon, small intestine, ileocecal junction, peri-hepatic, diaphragm, peritoneum, lymph nodes |
| P2 | 62 | III | High-grade | 530 | 293 | mutant IHC pattern | wild-type | uterus, uterine adnexa, rectum, omentum, colon, small intestine, ileocecal junction, peri-hepatic, peri-spleen, diaphragm, peritoneum, lymph nodes |
| P3 | 69 | IV | High-grade | 378 | 795 | mutant IHC pattern | wild-type | uterine adnexa, rectum, omentum, colon, small intestine, ileocecal junction, diaphragm, peritoneum, lymph nodes, hydrothroax |
| P4 | 67 | III | High-grade | 2426 | >1500 | mutant IHC pattern | wild-type | uterine adnexa, omentum, colon, small intestine, ileocecal junction, peri-hepatic, peri-spleen, diaphragm, peritoneum, lymph nodes |

**Table S1.** *Cont.*

| P5 | 65 | III | High-grade | 2038 | 721 | mutant IHC pattern | wild-type | uterus, uterine adnexa, rectum, omentum, colon, small intestine, ileocecal junction, peri-hepatic, diaphragm, peritoneum, lymph nodes |
| --- | --- | --- | --- | --- | --- | --- | --- | --- |
| P6 | 45 | III | High-grade | 714 | 470 | mutant IHC pattern | *BRCA1* deleterous mutation | uterus, uterine adnexa, rectum, omentum, colon, small intestine, ileocecal junction, peri-renal, peri-hepatic, diaphragm, peritoneum |
| P7 | 69 | III | High-grade | 3675 | >1500 | wild-type IHC pattern | wild-type | uterine adnexa, omentum, colon, peri-spleen, diaphragm, peritoneum |
| P8 | 58 | III | High-grade | 1037 | 233 | mutant IHC pattern | *BRCA1* deleterous mutation | uterus, uterine adnexa, rectum, omentum, colon, ileocecal junction, peri-hepatic, diaphragm, peritoneum, lymph nodes |
| P9 | 62 | III | High-grade | 2988 | 265 | mutant IHC pattern | *BRCA1* deleterous mutation | uterine adnexa, rectum, omentum, colon, small intestine, ileocecal junction, diaphragm, peritoneum, lymph nodes |
| P10 | 48 | III | High-grade | 7652 | 788 | mutant IHC pattern | wild-type | uterus, uterine adnexa, omentum, colon, ileocecal junction, peri-hepatic, diaphragm, peritoneum |
| P11 | 68 | III | High-grade | 1032 | 706 | mutant IHC pattern | wild-type | uterus, uterine adnexa, omentum, colon, small intestine, peritoneum, lymph nodes |
| P12 | 73 | III | High-grade | 501 | 1377 | mutant IHC pattern | wild-type | uterus, uterine adnexa, omentum, colon, peritoneum |

**Table S1.** *Cont.*

| P13 | 56 | III | High-grade | 2437 | 918 | mutant IHC pattern | wild-type | uterus, uterine adnexa, rectum, omentum, colon, small intestine, ileocecal junction, diaphragm, peritoneum |
| --- | --- | --- | --- | --- | --- | --- | --- | --- |
| P14 | 45 | III | High-grade | 1844 | 562 | mutant IHC pattern | wild-type | uterus, uterine adnexa, rectum, omentum, colon, small intestine, ileocecal junction, diaphragm, peritoneum |
| P15 | 59 | III | High-grade | 2325 | 1035 | mutant IHC pattern | wild-type | uterus, uterine adnexa, omentum, colon, ileocecal junction, peritoneum, lymph nodes |
| P16 | 49 | III | High-grade | 2100 | >1500 | mutant IHC pattern | *BRCA1* deleterous mutation | uterus, uterine adnexa, rectum, omentum, colon, small intestine, ileocecal junction, peri-renal, peri-hepatic, diaphragm, peritoneum, lymph nodes |
| P17 | 51 | III | High-grade | 787 | 616 | wild-type IHC pattern | wild-type | uterine adnexa, omentum, colon, small intestine, ileocecal junction, peritoneum, lymph nodes |
| P18 | 51 | III | High-grade | 520 | 532 | mutant IHC pattern | wild-type | uterus, uterine adnexa, omentum, colon, small intestine, peritoneum |
| P19 | 69 | III | High-grade | 3810 | >1500 | wild-type IHC pattern | *BRCA1* deleterous mutation | uterus, uterine adnexa, rectum, omentum, colon, ileocecal junction, peri-renal, peri-hepatic, peri-spleen, diaphragm, peritoneum, lymph nodes |
| P20 | 53 | III | High-grade | 3839 | 993 | mutant IHC pattern | wild-type | uterine adnexa, omentum, colon, peri-hepatic, peri-spleen, diaphragm, peritoneum, lymph nodes |
