# Supplementary figures and images for: Sphingosine kinase 1 contributes to the metastatic potential of epithelial ovarian cancer to the adipocyte-rich niche
Source: Exp Hematol Oncol. 2022 Nov 16;11:102. doi: 10.1186/s40164-022-00358-y (PMC9667684; doi:10.1186/s40164-022-00358-y)

**A**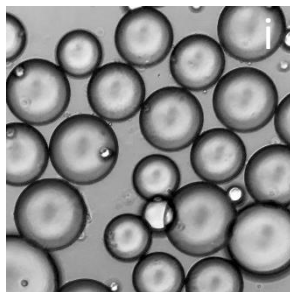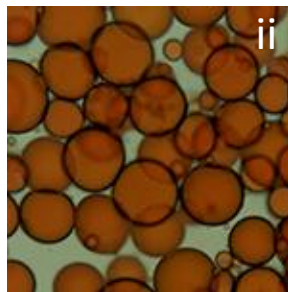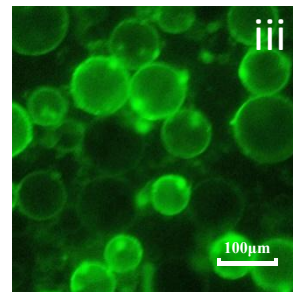**B**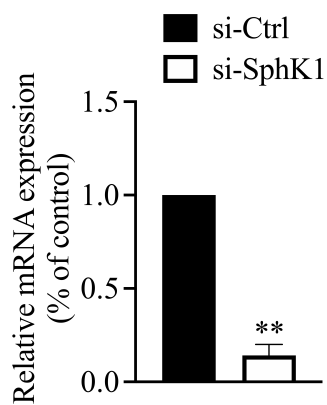**C**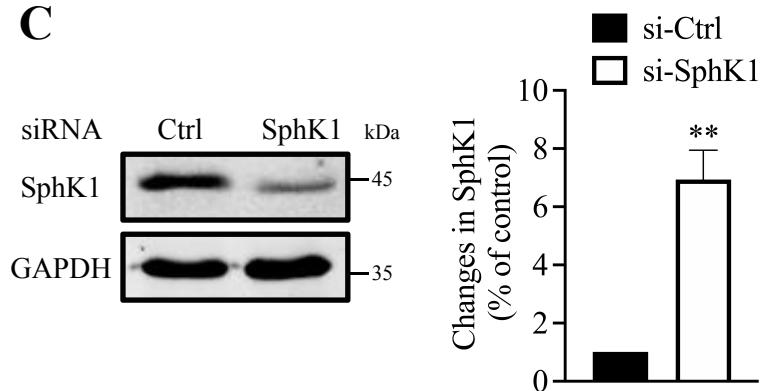**D**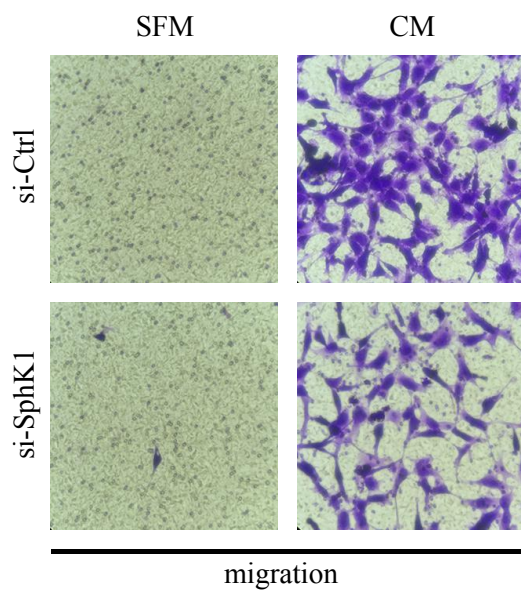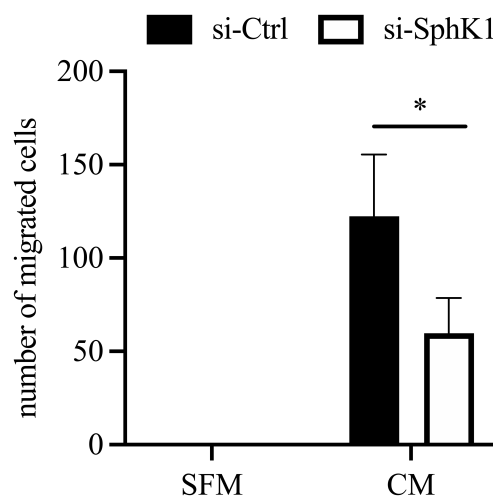**E**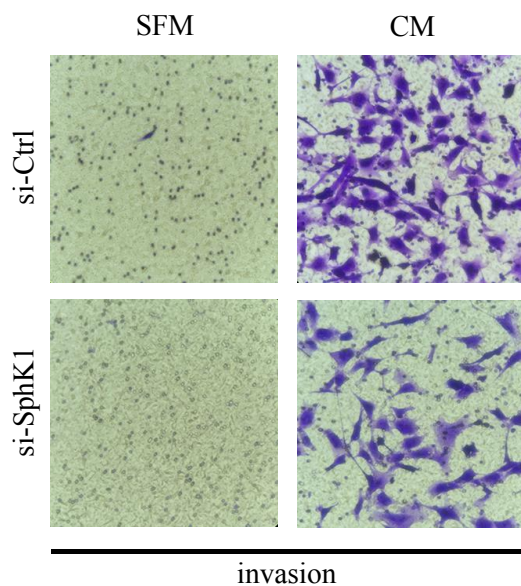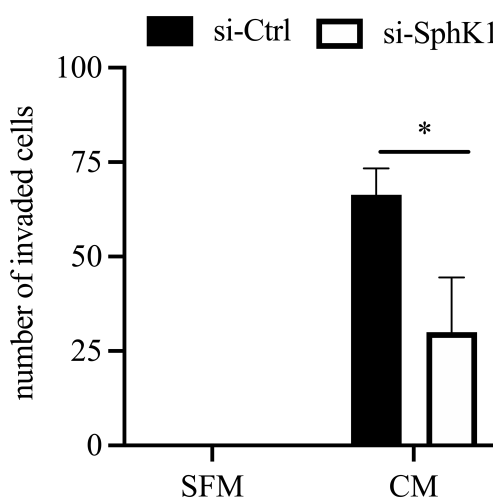

Supplement: Supplementary file 2 — Additional file 2: Fig. S1 Inhibition of SphK1 by siRNA suppressed adipocyte-induced metastasis of HEY cells. A Adipocyte isolation. Primary human omentum tissues were collected and mature adipocytes were extracted. Adipocytes were visualized by (i) microscopy, (ii) stained with Oil red O to confirm the extraction of mature adipocytes, and (iii) by fluorescence microscopy with calcein-AM to confirm viability. The scale bar represents 100 μm. B HEY cells were transfected with negative control siRNA or SphK1 siRNA. 24 h after transfection, mRNA level of SphK1 was determined by qRT-PCR and normalized to GAPDH. Data are mean ± SD (n = 3). **P < 0.01; two-tailed Student’s t test. C 48 h after transfection, protein level of SphK1 was determined by Western blot and normalized to GAPDH. Densitometric analysis of SphK1 was shown on the right. Data are mean ± SD (n = 3). **P < 0.01; two-tailed Student’s t test. D Representative images of migration assay (200×). Transfected HEY cells were serum starved overnight and migrated towards SFM or adipocyte CM. Migrated cells were photographed and calculated. Experiments have been carried out with adipocytes from 2 to 3 independent donors. Data are mean ± SD (n = 3). *P < 0.05; two-tailed Student’s t-test. E Representative images of invasion assay (200×). Transfected HEY cells were serum starved overnight and invaded towards SFM or adipocyte CM. Invaded cells were photographed and calculated. Experiments have been carried out with adipocytes from 2 to 3 independent donors. Data are mean ± SD (n = 3). *P < 0.05; two-tailed Student’s t test [file 40164_2022_358_MOESM2_ESM.pdf]

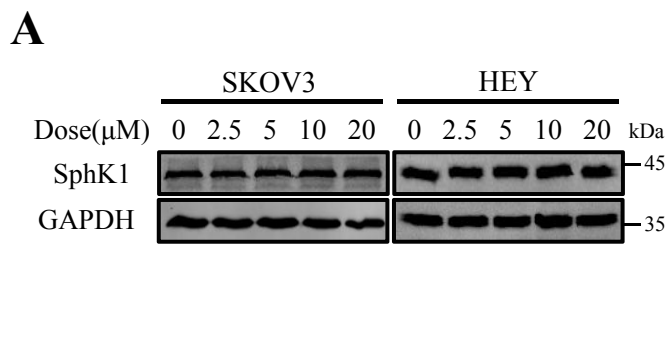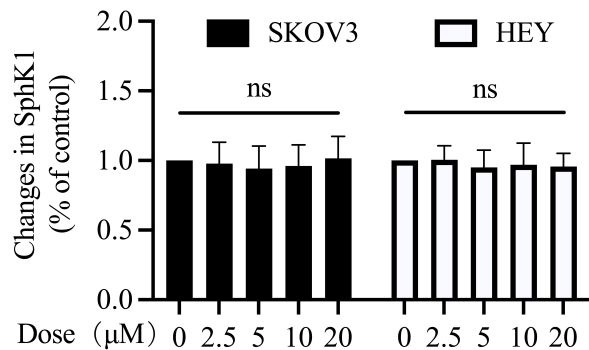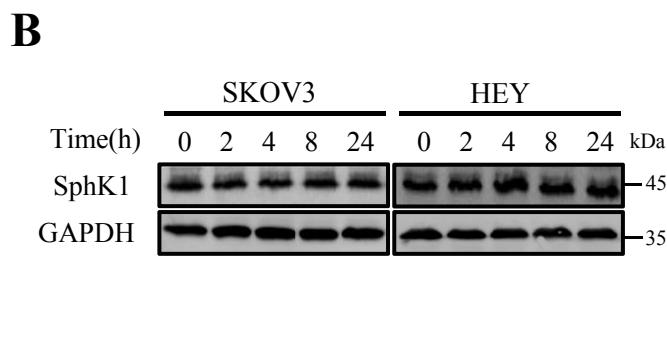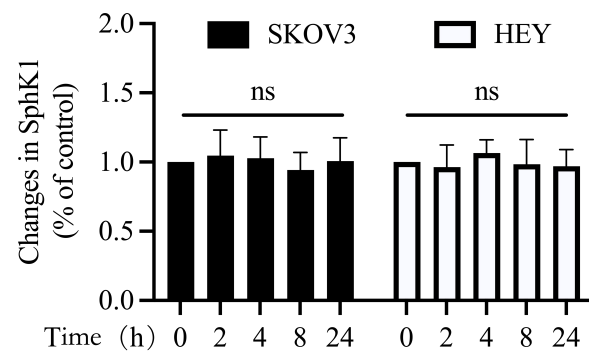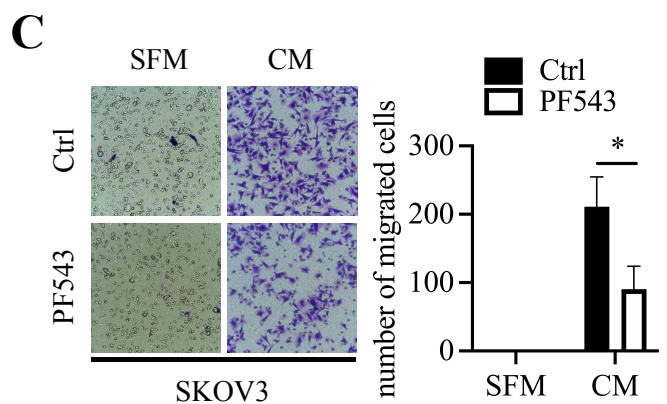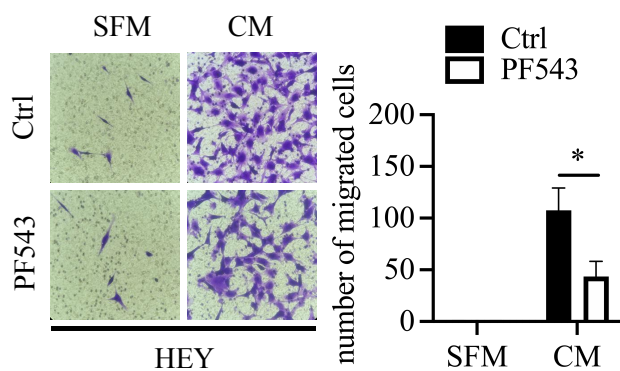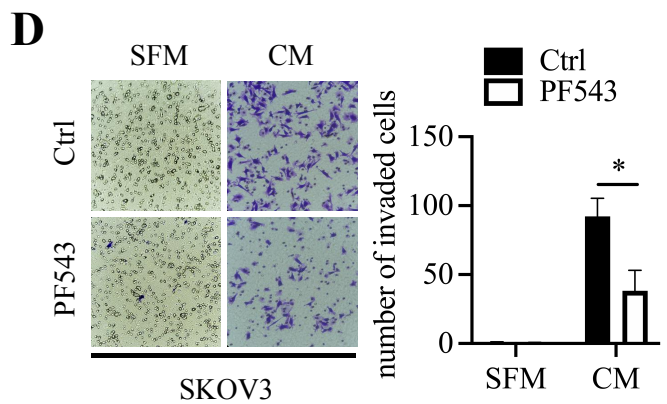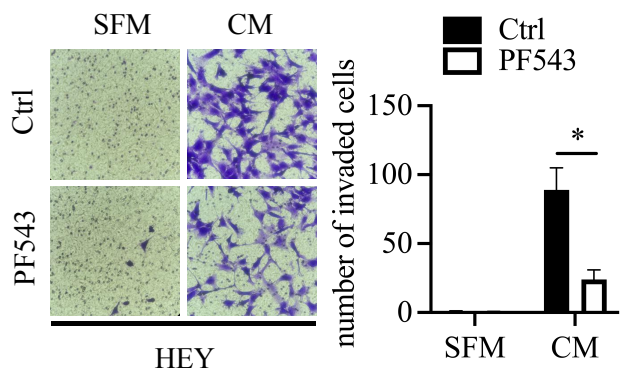

Supplement: Supplementary file 3 — Additional file 3: Fig. S2 SphK1 inhibitor PF543 suppressed adipocyte-induced metastasis of EOC cells. A Serum-starved SKOV3 and HEY cells were treated with increasing doses of PF543 as indicated for 2 h. Expression level of SphK1 was determined by Western blot and normalized to GAPDH. Densitometric analysis of SphK1 was shown on the right. Data are mean ± SD (n = 3). ns, no significance versus control; two-tailed Student’s t test. B Serum-starved SKOV3 and HEY cells were treated with 10 μM PF543 for the indicated time. Expression level of SphK1 was determined by Western blot and normalized to GAPDH. Densitometric analysis of SphK1 was shown on the right. Data are mean ± SD (n = 3). ns, no significance versus control; two-tailed Student’s t test. C Representative images of migration assay (200×). SKOV3 and HEY cells were serum starved overnight, pretreated with PF543 (10 μM) for 2 h, and then migrated towards SFM or adipocyte CM. Migrated cells were photographed and calculated. Experiments have been carried out with adipocytes from 2 to 3 independent donors. Data are mean ± SD (n = 3). *P < 0.05; two-tailed Student’s t test. D Representative images of invasion assay (200×). SKOV3 and HEY cells were serum starved overnight, pretreated with PF543 (10 μM) for 2 h, and then invaded towards SFM or adipocyte CM. Invaded cells were photographed and calculated. Experiments have been carried out with adipocytes from 2 to 3 independent donors. Data are mean ± SD (n = 3). *P < 0.05; two-tailed Student’s t test [file 40164_2022_358_MOESM3_ESM.pdf]

**A**

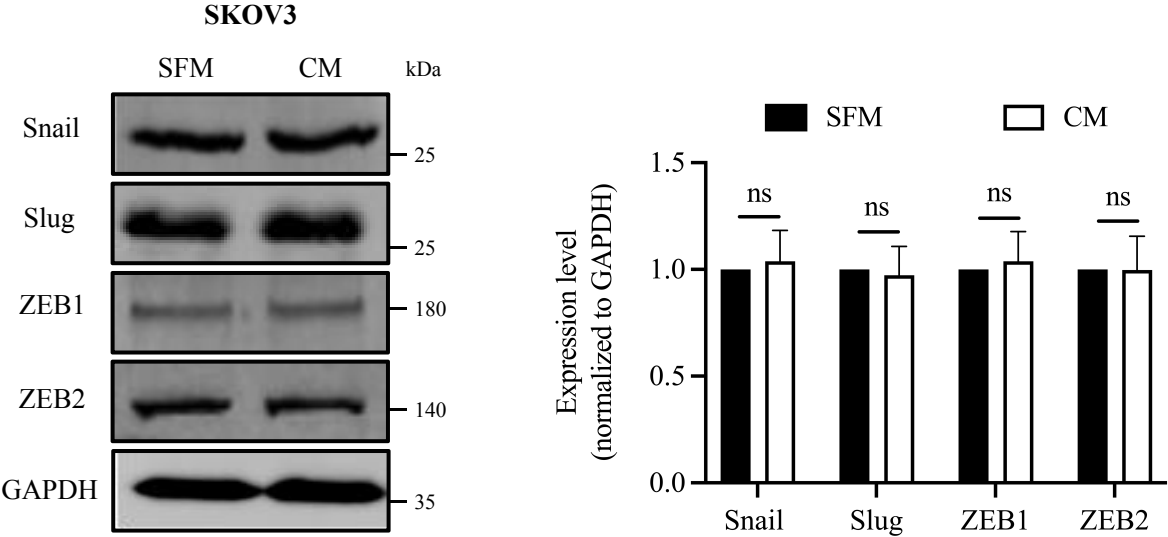

**B**

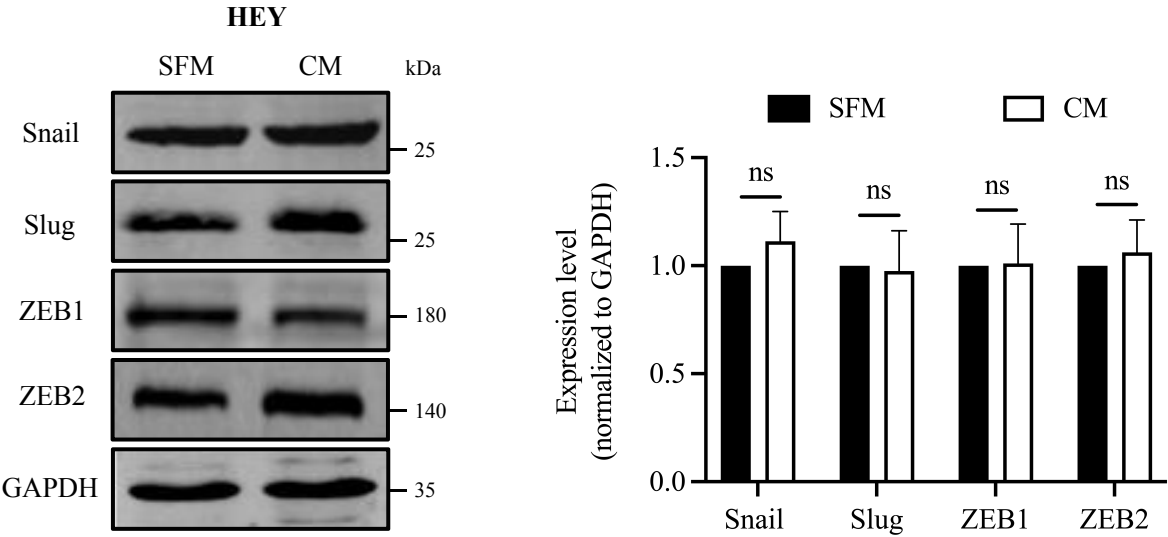

Supplement: Supplementary file 4 — Additional file 4: Fig. S3 Effect of adipocyte CM on the expression level of Snail, Slug, ZEB1 and ZEB2 in EOC cells. A SKOV3 cells were serum starved overnight and cultured in SFM or adipocyte CM for 48 h. Expression levels of Snail, Slug, ZEB1 and ZEB2 were determined by Western blot and normalized to GAPDH. Densitometric analyses were shown on the right. Experiments have been carried out with adipocytes from 2 to 3 independent donors. Data are mean ± SD (n = 3). ns, no significance; two-tailed Student’s t test. B HEY cells serum starved overnight and cultured in SFM or adipocyte CM for 48 h. Expression levels of Snail, Slug, ZEB1 and ZEB2 were determined by Western blot and normalized to GAPDH. Densitometric analyses were shown on the right. Experiments have been carried out with adipocytes from 2 to 3 independent donors. Data are mean ± SD (n = 3). ns, no significance; two-tailed Student’s t test [file 40164_2022_358_MOESM4_ESM.pdf]

**A**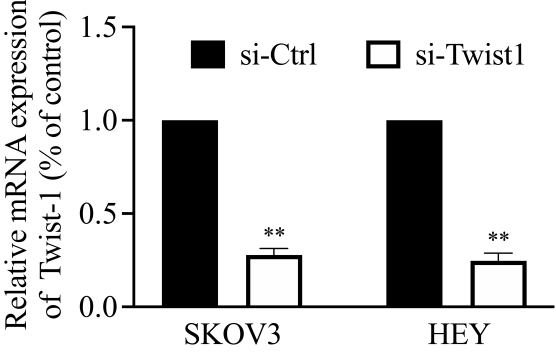**B**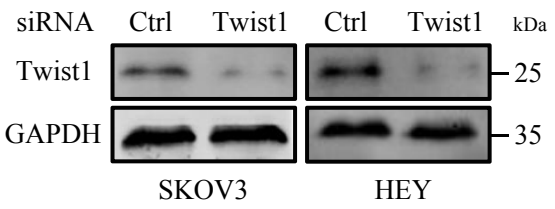**C**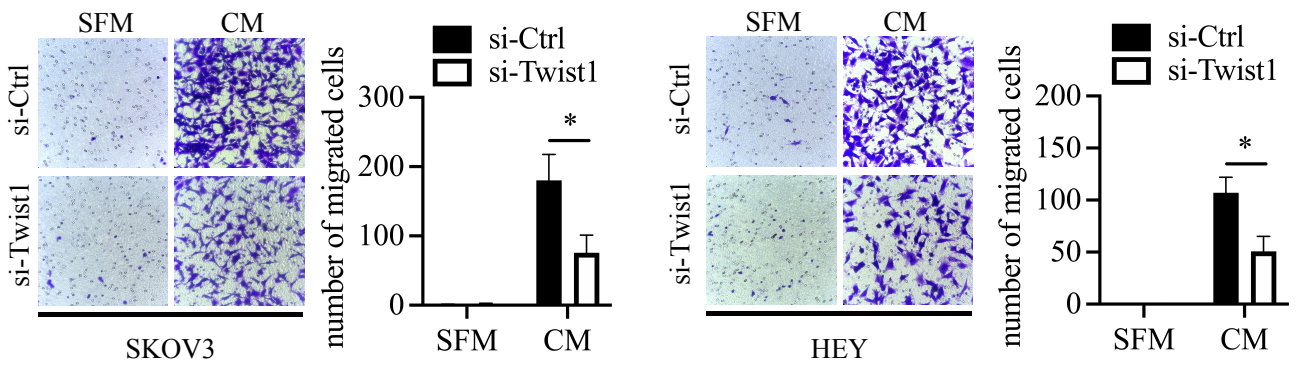**D**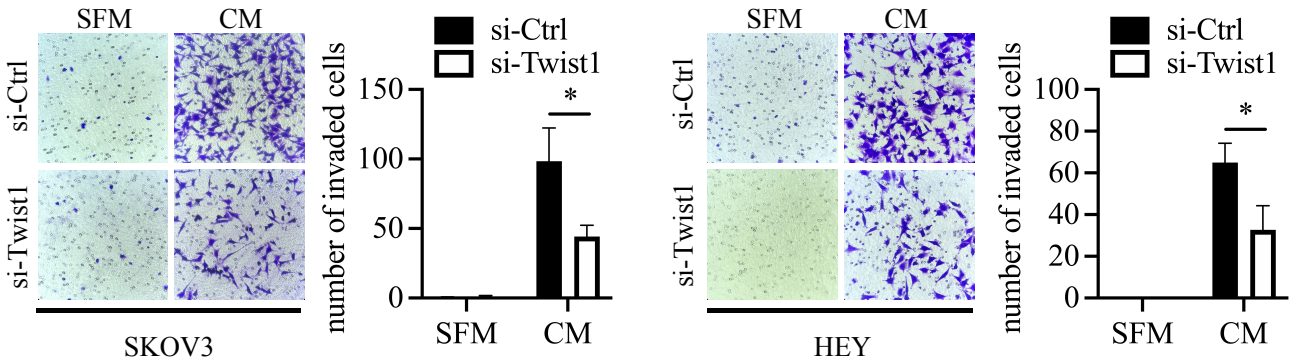

Supplement: Supplementary file 5 — Additional file 5: Fig. S4 Inhibition of Twist1 by siRNA suppressed adipocyte-induced metastasis of EOC cells. A SKOV3 and HEY cells were transfected with negative control siRNA (si-Ctrl) or Twist1 siRNA (si-Twist1). 24 h after transfection, mRNA level of Twist1 was determined by qRT-PCR and normalized to GAPDH. Data are mean ± SD (n = 3). **P < 0.01; two-tailed Student’s t-test. B 48 h after transfection, protein level of Twist1 was determined by Western blot and normalized to GAPDH. C Representative images of migration assay (200×). Transfected cells were serum starved overnight and migrated towards SFM or adipocyte CM. Migrated cells were photographed and calculated. Experiments have been carried out with adipocytes from 2 to 3 independent donors. Data are mean ± SD (n = 3). *P < 0.05; two-tailed Student’s t-test. D Representative images of invasion assay (200×). Transfected cells were serum starved overnight and invaded towards SFM or adipocyte CM. Invaded cells were photographed and calculated. Experiments have been carried out with adipocytes from 2 to 3 independent donors. Data are mean ± SD (n = 3). *P < 0.05; two-tailed Student’s t-test [file 40164_2022_358_MOESM5_ESM.pdf]

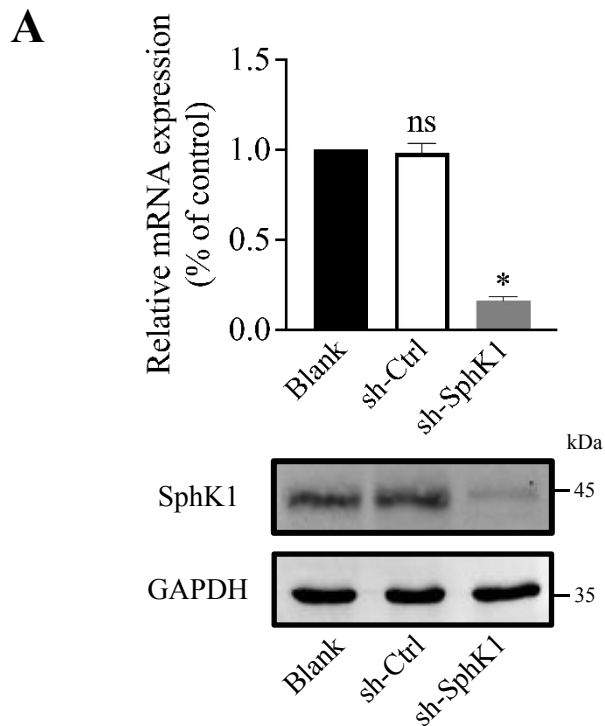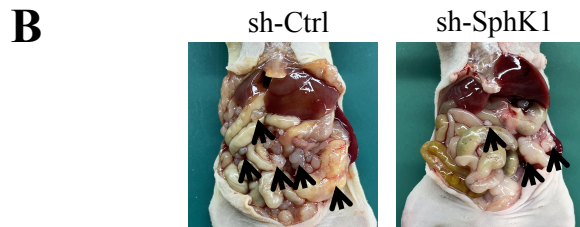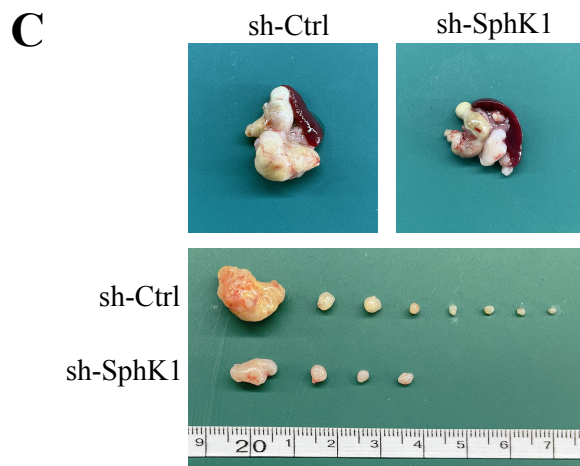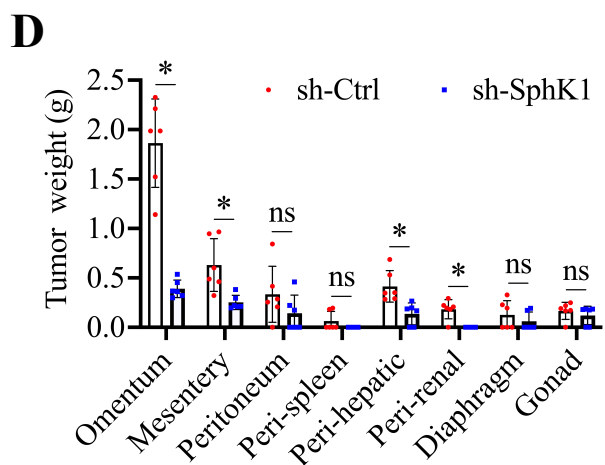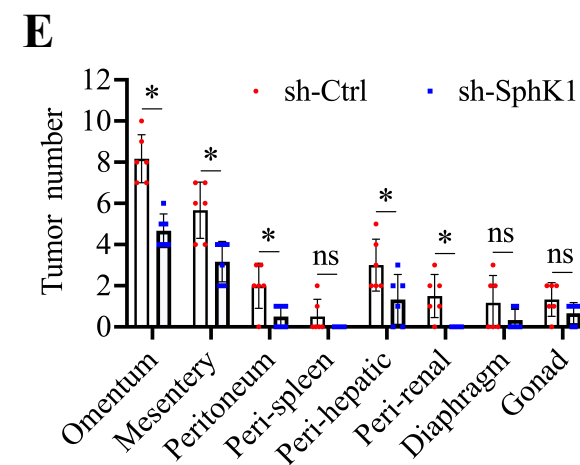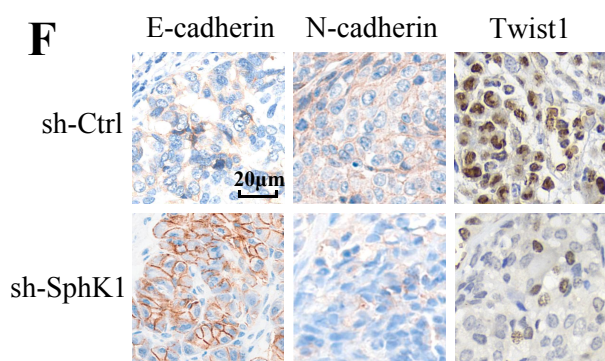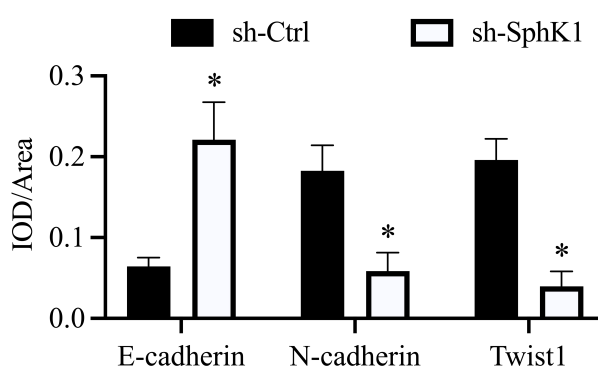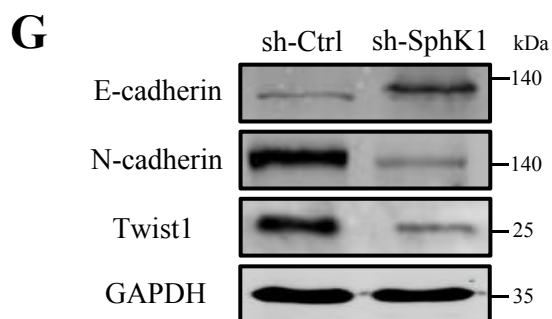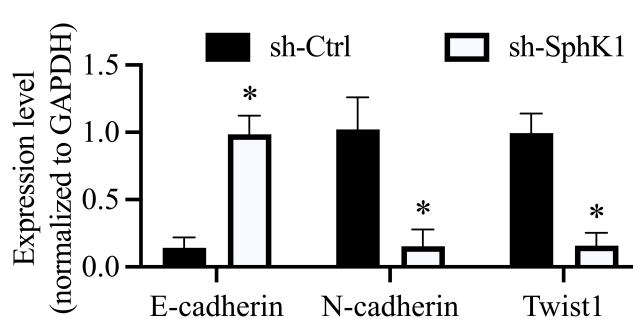

Supplement: Supplementary file 6 — Additional file 6: Fig. S5 Inhibition of SphK1 suppressed omental metastasis of EOC in vivo. A Expression of SphK1 in stable transfected SKOV3 cells tested by qRT-PCR and Western blot. GAPDH was used as a loading control. Data are mean ± SD (n = 3). *P < 0.05 versus Blank; ns, no significance versus Blank; two-tailed Student’s t-test. B Representative images of disseminated tumors in intraperitoneal EOC xenograft mouse models injected with Ctrl shRNA (sh-Ctrl) transfected or SphK1 shRNA (sh-SphK1) transfected SKOV3 cells. Black arrows indicated the location of the metastatic nodules. C Representative images of omental metastatic tumors in mouse models. D Quantification of tumor weight in different metastatic sites. Data are mean ± SD (n = 6). *P < 0.05 versus sh-Ctrl group; ns, no significance; two-tailed Student’s t test. E Quantification of tumor number in different metastatic sites. Data are mean ± SD (n = 6). *P < 0.05; ns, no significance; two-tailed Student’s t test. F IHC staining of E-cadherin, N-cadherin and Twist1 in omental metastatic tumor tissue of mouse models. The scale bar represents 20 μm. Statistical analysis of IOD/area was shown on the right. Data are mean ± SD (n = 6). *P < 0.05 versus sh-Ctrl group; two-tailed Student’s t-test. G Expression levels of E-cadherin, N-cadherin and Twist1 in omental metastatic tumor tissue were determined by Western blot and normalized to GAPDH. Densitometric analyses of E-cadherin, N-cadherin and Twist1 were shown on the right. Data are mean ± SD (n = 6). *P < 0.05 versus sh-Ctrl group; two-tailed Student’s t-test [file 40164_2022_358_MOESM6_ESM.pdf]
