# Supplementary material for: Sphingosine kinase 1 contributes to the metastatic potential of epithelial ovarian cancer to the adipocyte-rich niche
Source: Exp Hematol Oncol. 2022 Nov 16;11:102. doi: 10.1186/s40164-022-00358-y (PMC9667684; doi:10.1186/s40164-022-00358-y)
Supplement: Supplementary file 7 — Additional file 7: Supplementary Methods. [file 40164_2022_358_MOESM7_ESM.pdf]

Supplementary Methods

Sphingosine kinase 1 contributes to the metastatic potential of epithelial ovarian cancer to the adipocyte-rich niche

Chen Wang [1,2†], Taiyang Ye [1,2†], Wenjing Wang [1,2], Keqi Song [1,2], Jie Zhu[1,2*], Lan Dai[1,2*], Wen Di [1,2,3*]

[†]Chen Wang and Taiyang Ye have contributed equally to this work.

*Correspondence: diwen163@163.com; delta496@163.com; zhujie@renji.com.

[1] Department of Obstetrics and Gynecology, Ren Ji Hospital, School of Medicine, Shanghai Jiao Tong University, Shanghai 200127, China

[2] Shanghai Key Laboratory of Gynecologic Oncology, Ren Ji Hospital, School of Medicine, Shanghai Jiao Tong University, Shanghai 200127, China

[3] State Key Laboratory of Oncogenes and Related Genes, Shanghai Cancer Institute, Ren Ji Hospital, School of Medicine, Shanghai Jiao Tong University, Shanghai 200127, China

Reagents and antibodies

Antibodies against SphK1 (catalog: ab262697, rabbit) was purchased from Abcam (Cambridge, MA, USA). Antibody against GAPDH (catalog: G8795, mouse) was purchased from Sigma-Aldrich (St. Louis, MO, USA). Antibody against E-cadherin (catalog: 3195, rabbit), N-cadherin (catalog: 13116, rabbit) and Twist1 (catalog: 69366, rabbit) were purchased from Cell Signaling Technology (Danvers, MA, USA). Snail (catalog: 13099, rabbit), Slug (catalog: 12129, rabbit), ZEB1 (catalog: 66279, mouse) and ZEB2 (catalog: 14026, rabbit) were purchased from Proteintech (Wuhan, China). PF543 (catalog: HY-15425) was ordered from MedChemExpress (Monmouth Junction, NJ, USA).

Tissue samples

Primary tumor and omental metastasis tissue were collected from patients of EOC in Obstetrics and Gynecology Department of Renji Hospital, Shanghai Jiao Tong University, which included 20 cases of primary EOC (stage III-IV). These patients underwent surgical treatment, and none of them accepted preoperative chemotherapy. This study was approved by the Institutional Review Board of Shanghai Jiao Tong University, and all the patients provided informed consents.

Immunohistochemistry (IHC)

Tumor tissue from EOC patients or mouse models were embedded in paraffin and sliced. After blocking, the slides were incubated with each primary antibody.

Stained slices were observed and photographed with scanner. Statistical analysis of integrated optical density (IOD)/area were performed according to the guideline described in our previous study [1].

Cell lines and cell culture

Human EOC cell lines SKOV3 and HEY were purchased from American Type Culture Collection (Manassas, VA, USA), and cultured in DMEM (SH30022; Hyclone, South Logan, UT, USA) accompanied with 10% fetal bovine serum (10100147; Gibco, Grand Island, NY, USA) and 1% antibiotics (15140163; Gibco, Grand Island, NY, USA). Corresponding tests had been done to confirm that there was no mycoplasma or cross-contamination in these cells, and cells used in this study experienced less than 20 passages.

Isolation of human primary mature adipocytes

Isolation of human primary mature adipocytes was adapted from previous published protocols [2, 3]. Briefly, Fresh omental tissue was collected from patients with benign ovarian tumors. PBS (SH30256; Hyclone, South Logan, UT, USA) was used to wash the tissue for 3 times and then ophthalmic scissors was used to cut the tissue into chyle shape. The omental tissue was dissolved in digestion buffer composed of DMEM/F12 (SH-30023; Hyclone, South Logan, UT, USA), 0.1% BSA (735108; Roche, Basel, Switzerland) and 0.2% collagenase type I (SCR103; Sigma-Aldrich, St. Louis, MO, USA), and incubated at 37°C for 1 hour with shaking

violently every 20 minutes. Finally, the digested tissue was centrifuged at 300g for 10 minutes. Upper oil liquid was discarded and the middle milky white component was carefully collected, which was the mature adipocytes. Human primary mature adipocytes were cultured in DMEM/F12 supplemented with 10% fetal bovine serum and 1% antibiotics at 37°C under 5% CO_2 atmosphere. Oil red O stain kit (G1262; Solarbio, Beijing, China) was used to verify the lipid droplet composition in adipocytes, and calcein-AM stain kit (CA1630; Solarbio, Beijing, China) was used to confirm the viability of these mature primary adipocytes. Culture medium (CM) from human primary mature adipocytes were collected after these cells were cultured with the serum-free medium (SFM) for 24 hours. Adipocytes from six donors were used in this study. Experiments have been carried out with adipocytes from 2-3 independent donors and repeated at least for three times.

Transient [small interfering RNA (siRNA)] and stable [short hairpin RNA (shRNA)] transfection

The siRNA specifically targeting human SphK1 (5'-AAGAGCUGCAAGGCCUUGCCC-3') [4], the siRNA specifically targeting human Twist1 (5'-GCAAGAUUCAGACCCUCAATT-3') [5] and the scrambled control siRNA (5'-AAUUCUCCGAACGUGUCACGU-3') were synthesized from GenePharma (Shanghai, China). Lipofectamine 2000 (Invitrogen, Carlsbad, CA, USA) was used to transfect the siRNA duplexes according to the manufacturer's protocol. 24 hours after transfection, the expression levels of targeted genes were detected by

qRT-PCR. 48 hours after transfection, the levels of targeted proteins were tested by Western blot. The shRNA specifically targeting human human SphK1 (5'-AAGAGCUGCAAGGCCUUGCCC-3') and the non-targeting negative control shRNA (5'-AAUUCUCCGAACGUGUCACGU-3') were synthesized from GenePharma. SKOV3 cell line were transfected with lentiviral vectors at a multiplicity of infection (MOI) =5. To establish SKOV3 cells stably down-regulating SphK1, transfected cells were selected by culturing in medium with puromycin. Single colonies of stable transfectants were isolated and expanded. The expression level of targeted gene was detected by qRT-PCR, and the level of targeted protein was tested by Western blot.

qRT-PCR

Isolation of total RNA was performed by TRIzol Reagent (Invitrogen, Carlsbad, CA, USA). Reverse transcription was performed by Primescript^{TM} RT Reagent Kit (Takara, Tokyo, Japan) according to the manufacturer’s protocol. Expression levels of mRNA were measured by SYBR Green RT-PCR and calculated by $2^{-\Delta\Delta Ct}$ method. Primers for specific genes were synthesized by Sangon (Shanghai, China). The following sequences were primers used in this study: SphK1, 5’-CATTATGCTGGCTATGAGCAG-3’ (forward) and 5’-GTCCACATCAGCAATGAAGC-3’ (reverse) [6]; E-cadherin, 5’-ATTTTTCCCTCGACACCCGAT-3’ (forward) and 5’-TCCCAGGCGTAGACCAAGA-3’ (reverse); N-cadherin,

5’-TCAGGCGTCTGTAGAGGCTT-3’ (forward) and 5’-ATGCACATCCTTCGATAAGACTG-3’ (reverse); Twist1, 5’-GTCCGCAGTCTTACGAGGAG-3’ (forward) and 5’-GCTTGAGGGTCTGAATCTTGCT-3’ (reverse); GAPDH, 5’-TGCACCACCAACTGCTTAGC-3’ (forward) and 5’-GGCATGGACTGTGGTCATGAG-3’ (reverse).

Western blot

Western blotting was performed as previously described [7]. Briefly, RIPA lysis buffer (P0013; Beyotime, Nantong, Jiangsu, China) added with protease inhibitor cocktail (ST506; Beyotime, Nantong, Jiangsu, China) was used to splitted EOC cells or tumor tissues after indicated treatments. BCA reagent (P0012; Beyotime, Nantong, Jiangsu, China) was used to measure the protein concentration. After denaturing at 100°C for 10 minutes, the protein was added into 10% standard SDS gel for electrophoresis until the protein of different molecular weight was separated. Then the protein was transferred to a PVDF membrane (R1CB73920; Millipore, Billerica, MA, USA), which has been activated by methanol. PVDF membrane with protein on it was blocked using 5% bovine serum albumin for 1 hour at room temperature. The membranes were probed with the indicated primary antibodies at 4°C for the whole night. TBST was used to wash the PVDF membrane for 3 times. After washing, the membrane was incubated with indicated secondary antibodies at room temperature for 1 hour. Indicated proteins on the membrane were visualized by Odyssey Film Scanner.

The relative gray scale of each protein was analyzed using the Image-J software.

Cell Migration assay

EOC cells (80,000) which starved overnight were added to the upper chamber (3422; Corning Incorporated, Corning, NY, USA) and allowed to migrate for 8 hours at 37°C. SFM and adipocyte CM were used as chemoattractant and added to the lower chamber. After treatment, 4% paraformaldehyde was used to fix EOC cells. Cells in the upper chamber were carefully removed with cotton swabs, while cells in the lower chamber were stained with crystal violet. Numbers of migrated cells in 5 fields per well in triplicate were quantified.

Matrigel invasion assay

Invasion ability of EOC cells was tested by Transwell chambers pre-coated with matrigel (356230; Corning Incorporated, Corning, NY, USA). EOC cells (80,000) which starved overnight were added to the upper chamber and allowed to invade for 24 hours at 37°C. SFM or adipocyte CM was added to the lower chamber. After treatment, 4% paraformaldehyde was used to fix EOC cells. Cells in the upper chamber were removed and cells in the lower chamber were stained with crystal violet. The invaded cells in 5 fields per well in triplicate were counted.

Animal studies

All animal experiments were carried out following the National Institutes of Health

(NIH) Guide for the Care and Use of Laboratory Animals 2018 and approved by Shanghai Jiao Tong University School of Medicine. The experimental protocols were approved by the Institutional Animal Care and Use Committee of Shanghai Jiao Tong University School of Medicine. Female BALB/c nu/nu mice aged 6 weeks were purchased from the Chinese Academy of Sciences. To establish intraperitoneal transplantation models, mice were intraperitoneally injected with 5×10^6 SKOV3 cells or stably transfected SKOV3 cells. Mice injected with 5×10^6 SKOV3 cells were randomly divided into control group and PF543 treatment group (n=6 in each group) 7 days after injection. Mice in the PF543 treatment group were injected with PF543 (5mg/kg) intraperitoneally, 2 times per week for 4 weeks. Mice in the control group were injected intraperitoneally with an equivalent amount of DMSO in PBS for 4 weeks. 35 days after injection of tumor cells, the mice were sacrificed. The weight and the number of metastatic tumors were calculated. The largest omentum metastatic tumor from each mouse was used for Western blot and IHC testing.

Statistical analysis

Statistical analysis was performed using the SPSS software (IBM Corporation, Armonk, NY, USA). The values were presented as the mean ± SD and analyzed by t-test ($p<0.05$ was considered significant).

Supplementary References

1. Dai, L., et al., Sphingosine kinase 1/sphingosine-1-phosphate (S1P)/S1P receptor axis is involved in ovarian cancer angiogenesis. Oncotarget, 2017. 8(43): 74947-74961.
2. Miranda, F., et al., Salt-Inducible Kinase 2 Couples Ovarian Cancer Cell Metabolism with Survival at the Adipocyte-Rich Metastatic Niche. Cancer Cell, 2016. 30(2):273-289.
3. Nieman, K.M., et al., Adipocytes promote ovarian cancer metastasis and provide energy for rapid tumor growth. Nature Medicine, 2011. 17(11): 1498-U207.
4. Dai, L., et al., Activation of SphK1 by adipocytes mediates epithelial ovarian cancer cell proliferation. J Ovarian Res, 2021. 14(1): 62.
5. Yang, J., et al., Twist, a master regulator of morphogenesis, plays an essential role in tumor metastasis. Cell, 2004. 117(7): 927-39.
6. Dai, L., et al., Sphingosine kinase (SphK) 1 and SphK2 play equivalent roles in mediating insulin's mitogenic action. Mol Endocrinol, 2014. 28(2): 197-207.
7. Dai, L., et al., Inhibition of sphingosine kinase 2 down-regulates ERK/c-Myc pathway and reduces cell proliferation in human epithelial ovarian cancer. Ann Transl Med, 2021. 9(8): 645.
